# Two Families With Amyotrophic Lateral Sclerosis Founder Mutation TARDBP p.G298S in Hong Kong

**DOI:** 10.7759/cureus.95667

**Published:** 2025-10-29

**Authors:** Man Kwan Yip, Man Au Yeung, Wing Tat Poon

**Affiliations:** 1 Department of Clinical Pathology, Pamela Youde Nethersole Eastern Hospital, Hong Kong, HKG; 2 Department of Medicine, Pamela Youde Nethersole Eastern Hospital, Hong Kong, HKG

**Keywords:** diagnostic genetic testing, familial amyotrophic lateral sclerosis, founder mutation, genetic landscape, tardbp

## Abstract

Amyotrophic lateral sclerosis (ALS), which is characterized by progressive deterioration of upper and lower motor neurons resulting in severe muscle atrophy, respiratory failure, and death, is a rare and fatal neurodegenerative disease.* TARDBP* p.G298S was recently identified as a founder mutation in southern Chinese. This article first presented case summaries of three ALS patients: two families with *TARDBP* p.G298S presenting with heterogeneous clinical phenotypes, including a case with an unusual extraocular muscle onset. A review of* TARDBP* p.G298S cases reported worldwide was conducted, surveying the age and site of onset, disease duration, and motor neuron involvement. Finally, an overview of genetic mutations reported locally for ALS was presented, showing that *TARDBP* p.G298S is a common mutation detected in this locality. This article highlighted the distinct clinical manifestations and genetic background in ALS patients and will be useful for developing genetic screening and counseling strategies in Hong Kong and southern China.

## Introduction

Amyotrophic lateral sclerosis (ALS) is a progressive and fatal neurodegenerative disease that affects both upper and lower motor neurons, leading to muscle weakness and respiratory failure. It is the most common form of motor neuron disease, characterized by the degeneration of both motor neurons. The worldwide incidence of ALS ranges from 0.3 to 7.0 per 100,000 people annually; in Hong Kong, it is estimated at 0.6 per 100,000 [1,2]. A recent local ALS registry has identified around 200 patients, revealing that it takes an average of one year for diagnosis, during which many patients become severely disabled. The rapid progression of ALS highlights the need for improved patient care and diagnostic methods. Diagnosis is primarily clinical, and the Gold Coast criteria offer a simplified approach that shows comparable effectiveness to previous criteria, potentially reducing delays in diagnosis and access to clinical trials.

About 5-10% of ALS cases are familial (fALS), while 90-95% are sporadic (sALS) [1]. Only 10-15% of cases are linked to known genetic mutations. The chance of a positive genetic test is around 70% for fALS and 10% for sALS [3]. Most fALS cases are inherited in an autosomal dominant manner, with over 30 genes associated with the disease. Genetic mutations differ by population. In Europeans, the most common mutation is the pathogenic *C9orf72* expansion, followed by pathogenic variants in *SOD1*, *TARDBP*, and *FUS*. In Asian populations, *SOD1* is the most common mutated gene, followed by *FUS*, *C9orf72*, and *TARDBP*. Different screening strategies are thus recommended: Europeans should first test for *C9orf72* expansion, while Asians should prioritize *SOD1 *mutations [3,4]. Meanwhile, *TARDBP* p.G298S was recently identified as a founder mutation in southern Chinese [5]. We identified the p.G298S mutation in *TARDBP* in both families. Two families with *TARDBP* p.G298S presenting with heterogeneous clinical phenotypes, including a case with an unusual initial symptom involving the extraocular muscles (EOMs) that facilitated presymptomatic genetic diagnosis, were referred to our laboratory. A clinical review of *TARDBP* p.G298S cases reported worldwide and an overview of genetic mutations reported locally for ALS were presented, suggesting that *TARDBP* p.G298S may be a common mutation in this locality.

## Case presentation

**Figure 1:**
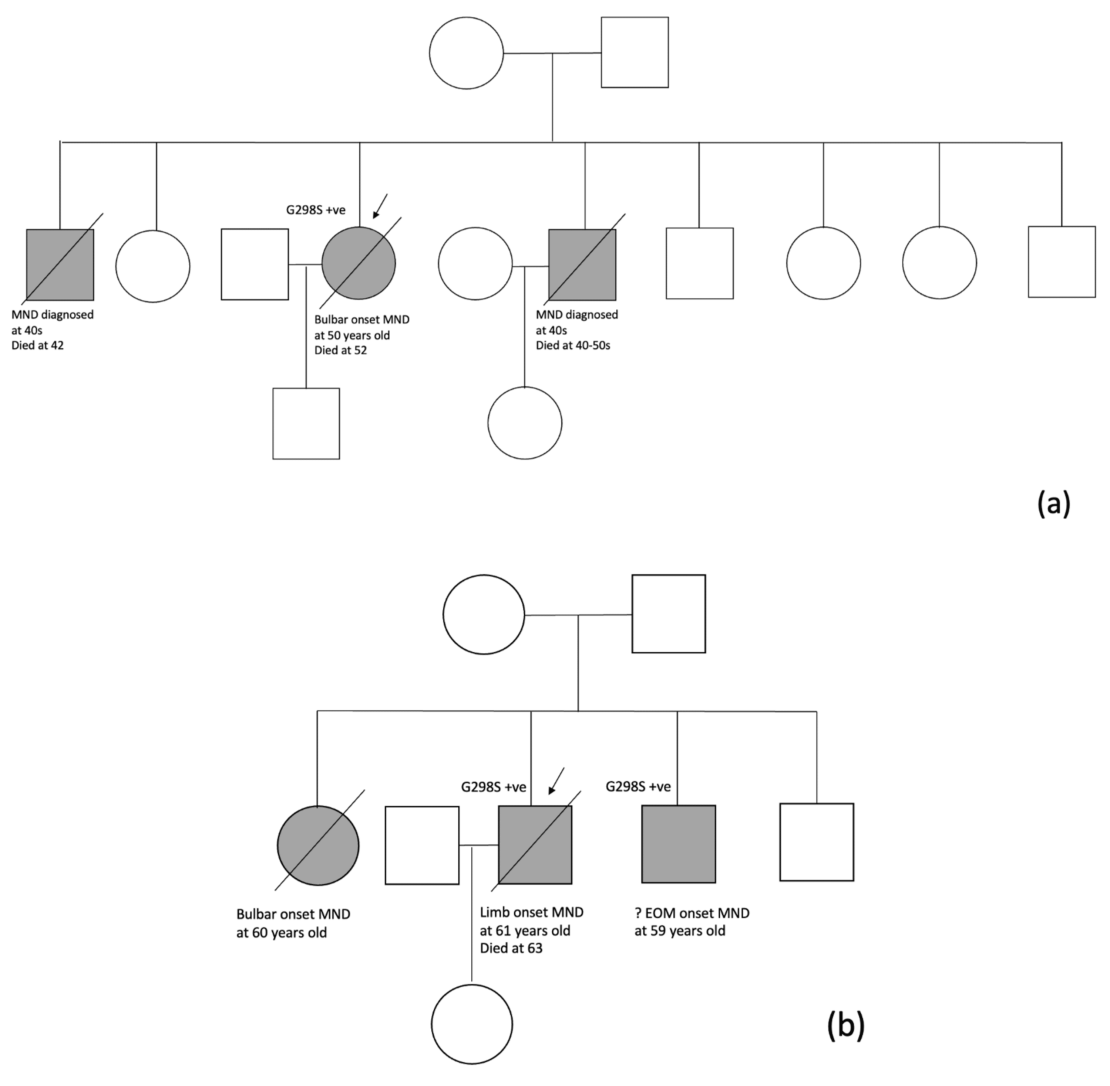
Pedigrees of the two families with fALS associated with TARDBP p.G298S mutation: (a) family 1 and (b) family 2 (a) In family 1, the female proband had bulbar onset MND at 50 years old and eventually passed away at age 52, while her elder and younger brothers were diagnosed with MND in their 40s. Other family members were reported to be asymptomatic. (b) In family 2, the male proband had limb onset of MND at 61 years old and succumbed at age 63. In comparison, his younger sister and younger brother were diagnosed with bulbar onset MND and suspected EOM onset MND at 60 and 59 years old, respectively. The proband also had four half-siblings, but contact was lost, and they were not included in the pedigree. Other family members were reported to be asymptomatic. fALS: familial amyotrophic lateral sclerosis, MND: motor neuron disease, EOM: extraocular muscle

Clinical details of two families with fALS associated with the *TARDBP* p.G298S mutation were presented as follows (Figure 1, Table 1).

**Table 1:** Clinical features of the three cases reported (U/L)MN: upper/lower motor neuron, UL: upper limb, LL: lower limb, EOM: extraocular muscle

Case	Presence of family history?	Mutation	Gender	Age of onset (years)	Time from onset to diagnosis made (months)	Disability at diagnosis	Drug treatment started	Age of death (years)	Region of onset	Symptoms spread to bulbar/spinal region?	Type of MN predominance	Presence of cognitive impairment?	Disease duration (months)
1	Yes	Heterozygous *TARDBP* G298S	Female	50	3	Bulbar problem	No	52	Bulbar	Yes	LMN	No	15
2.1	Yes	Heterozygous *TARDBP* G298S	Male	61	1	Weakness in the limbs and dyspnea	Yes	63	Spinal (UL+LL)	Yes	LMN	No	17
2.2	Yes	Heterozygous *TARDBP* G298S	Male	59	6	Binocular diplopia	Yes	/	?EOM	No	LMN	No	>16

Family 1

In July 2020, a 50-year-old woman with a strong family history of motor neuron disease was referred for slurred speech, hoarseness, choking, and shortness of breath that developed over three months. Her elder and younger brothers were diagnosed with motor neuron disease in their 40s and died of respiratory failure in their 40s and 50s. At the same time, other family members did not report a history of neurological diseases. She had no cognitive issues, with a normal Hong Kong version of the Montreal Cognitive Assessment (HK-MoCA) result. Exams showed bulbar speech and muscle twitching in her tongue and limbs, especially her left arm. An MRI revealed cervical spine degeneration, but no clear cause for her symptoms. A nerve conduction study showed a slight decrease in compound muscle action potential amplitude over the bilateral median and peroneal nerves, which may indicate mild axonal neuropathy. Electromyography showed suspicious fasciculation and active denervation in small hand muscles, with features of reinnervation also noted in left upper limb muscles. There was also a reduced recruitment ratio over the proximal rectus muscle and the tibialis anterior muscle in the lower limbs. The overall findings were compatible with motor neuron disease. Genetic testing identified a heterozygous c.892G>A (p.G298S) pathogenic variant in the *TARDBP* gene. Eight months later, her breathing and swallowing worsened, requiring feeding support and noninvasive ventilation. By June 2021, she could not move her limbs against gravity or stick out her tongue. Eventually, she passed away 15 months after her symptoms began, at age 52.

Family 2

A 61-year-old man with diabetes, hypertension, ischemic heart disease, and sleep apnea experienced progressive weakness in his right arm and both legs starting in February 2021. He had three full siblings, and his younger sister was diagnosed with bulbar-onset ALS at 60 years old. He also had four half-siblings, but contact was lost. No neurological diseases were reported in his father and mother, yet a strong family history of nasopharyngeal cancer was reported on his maternal side. Nerve conduction study and electromyography showed preserved sensory nerve action potentials, with evidence of widespread denervation changes in the right upper and bilateral lower limb muscles, suggestive of motor neuron disease, while brain scans were normal. He began taking riluzole and felt some improvement. In April 2021, he developed breathing difficulties, and by June, he had nasal speech and dysphagia. Physical exams showed bulbar speech, flaccid muscle tone, and generalized hyporeflexia. He started using noninvasive ventilation at home in August. Tests showed positive anti-acetylcholine receptor antibodies, and he received a course of intravenous immunoglobulin but did not improve significantly. Myasthenia gravis was considered unlikely given severe muscle wasting and stable motor function, and anticholinesterase agents were not initiated. Genetic testing revealed the *TARDBP* p.G298S variant. By October 2021, his breathing had worsened. In March 2022, he was hospitalized for COVID-19 and developed pneumonia, leading to respiratory failure. He passed away 17 months after his symptoms began, in August 2022, at age 63.

In December 2023, the 59-year-old younger brother of the proband was referred for binocular diplopia at extreme lateral gaze, with a gradual onset over 5 months. There were no prior flu-like symptoms, nausea, vomiting, headache, eye pain, or tinnitus. He had a history of hypertension, impaired fasting glucose, ischemic heart disease, renal stones, and frozen shoulder. His limb strength was normal, but he was suspected of having right abducens nerve palsy, along with hyporeflexia that was initially thought to be due to diabetic neuropathy. The nerve conduction test was unremarkable, and electromyography showed polyphasic motor waves in his right tibialis anterior, deltoid, biceps, and first dorsal interosseous muscles, which were suggestive of reinnervation changes with a chronic denervation pattern. Brain and spine MRIs were normal, and tests for anti-acetylcholine receptor antibodies were negative. Genetic testing confirmed the presence of the familial *TARDBP* p.G298S variant. Within two weeks, he started treatment with riluzole for likely early *TARDBP*-related ALS in March 2024. The option of self-financed edaravone was also discussed. A week later, he reported no more double vision and full movement in his eye muscles. The patient remained asymptomatic at the latest follow-up in December 2024, with no definite disease progression on nerve conduction studies and electromyography. Riluzole is currently the only drug shown to improve survival in ALS, especially for patients with symptoms lasting less than five years and good lung function. Edaravone, a free radical scavenger, and sodium phenylbutyrate-taurursodiol, which is not yet available in Hong Kong, are also indicated for ALS patients. Symptomatic management otherwise is the mainstay of treatment in ALS.

Materials and methods

Clinical information for two probands and their family members from families 1 and 2 was collected from the electronic hospital database. In family 2, the proband's younger brother was also referred for family genetic screening.

Genetic testing was performed on all coding exons and the 10-base-pair flanking regions of *SOD1*, *FUS*, and *TARDBP* for the two probands. At the same time, only the familial variant was targeted in the younger brother of the proband in family 2. Next-generation sequencing (NGS) was performed on the proband from family 1, while Sanger sequencing was performed on the proband and his younger brother in family 2. Supplementary ExpansionHunter analysis of PCR-free NGS data would be performed (which was not done in these three patients) to estimate the size of the *C9orf72* repeat expansion, only if no mutations were detected in the *SOD1*, *FUS*, or *TARDBP* genes, due to the rarity of this mutation in our locality.

For Sanger sequencing, DNA from peripheral blood was extracted using the Qiagen QIAamp DNA Blood Mini Kit (Qiagen, Hilden, Germany) according to the manufacturer’s instructions. Target exons were amplified from extracted genomic DNA by PCR. Sanger sequencing was performed using the BigDye Terminator v1.1 Cycle Sequencing Kit (Applied Biosystems, CA, USA) and an ABI 3500 genetic analyzer.

For NGS, DNA extraction and purification were performed on the submitted sample using the Qiagen QIAamp DNA Blood Mini Kit (Qiagen, Hilden, Germany), and the sample was enriched using the MGIEasy FS PCR-free DNA Library Prep (MGI Tech, Shenzhen, China) according to the manufacturer’s instructions. One hundred base-pair, paired-end (PE100) DNA sequencing was performed on a DNBSEQ-G400 sequencer (MGI Tech, Shenzhen, China). Variant calling and filtering were performed using an in-house bioinformatics pipeline. In general, all target regions were sequenced with 20x or greater coverage, with a Phred-scaled quality score of 20 or above, and a mapping quality score of 20 or above, and quality-checked using SAMtools (version 0.3.3) and a custom in-house Python script. Exceptionally, target regions with sequencing coverage below the quality standard would be individually sequenced using Sanger sequencing.

## Discussion

In Asian populations, *SOD1* is the most common mutated gene, followed by *FUS*, *C9orf72*, and *TARDBP* (Table 2). Here, we presented two families with *TARDBP*-related ALS, which is thus expected to be seen less frequently in the local cohort.

**Table 2:** Mutation rate of four major ALS-related genes ALS: amyotrophic lateral sclerosis, fALS: familial amyotrophic lateral sclerosis, sALS: sporadic amyotrophic lateral sclerosis [4]

Mutation	European		Asian	
	fALS	sALS	fALS	sALS
*C9orf72* repeat expansions	33.7%	5.1%	2.3%	0.3%
SOD1	14.8%	1.2%	30.0%	1.5%
TARDBP	4.2%	0.8%	1.5%	0.2%
FUS	2.8%	0.3%	6.4%	0.9%

*TARDBP* p.G298S cases reported worldwide

Most ALS cases involve aggregates of the TDP-43 protein in cells. TDP-43, encoded by the *TARDBP* gene, is important for RNA processing and protein synthesis in dendrites. Evidence suggests it may act like a prion, leading to protein misfolding [6]. There is ongoing debate over whether *TARDBP* mutations cause ALS via loss-of-function (nuclear depletion) or gain-of-function (cytoplasmic aggregation) mechanisms [7]. Most mutations, including the p.G298S mutation, which is mainly found in Southern Chinese, occur in exon 6 and affect a key region involved in motor neuron degeneration [5,6].

A summary of all cases (n=40) with the *TARDBP* p.G298S mutation reported globally shows it is primarily found in Eastern Asia among the ethnic Chinese and Japanese populations, with one case in a Caucasian patient in the U.S. (Table 3) [1,5,8-14]. The mutation was first reported in a Chinese family in the U.S. in 2008, where all five patients quickly progressed to quadriparesis and respiratory failure within one to four years, regardless of onset type [9]. In 2022, a study identified the mutation's founder effect in 16 patients from Southern China (eight with familial ALS and eight with sporadic ALS), most of whom were from Guangdong and Guangxi provinces. Carriers of the p.G298S mutation typically exhibited limb onset and had a short average survival of about 18.3 months. About 60% of patients with the *TARDBP* p.G298S mutation are male or have a family history of ALS. None of the patients showed clear cognitive impairment, despite TDP-43 inclusions being linked to frontotemporal dementia (FTD) [5]. The most common onset location is the spinal region, typically starting in the upper limbs, with bulbar onset occurring in 30% of cases.

**Table 3:** Summary of 40 cases with TARDBP p.G298S detected worldwide. Cases reported in the literatures (patients 1-34) and in this case series (patients 35-40) are detailed with the patients' country of origin, ethnicity, type of ALS (sALS/fALS), family history, gender, age of onset and death, region of onset, progression of symptoms to bulbar or spinal region, type of MN predominance (UMN/LMN), cognitive impairment, and disease duration. # Genetic testing was only performed in other family members, but not this patient [1,5,8-14]. sALS: sporadic amyotrophic lateral sclerosis, fALS: familial amyotrophic lateral sclerosis, (U/L)MN: upper/lower motor neuron, UL: upper limb, LL: lower limb, EOM: extraocular muscle

Patient	Country of origin	Ethnicity	sALS/fALS	Positive family history?	Gender	Age of onset (years)	Age of death (years)	Region of onset	Symptoms spread to bulbar/spinal region?	Type of MN predominance	Presence of cognitive impairment?	Disease duration (months)
1	USA	Chinese	fALS#	Yes	Male	47	48	/	/	/	No	12
2	USA	Chinese	fALS#	Yes	Male	48	49	/	/	/	No	12
3	USA	Chinese	fALS	Yes	Female	60	62	Bulbar	Yes	UMN/LMN	No	24
4	USA	Chinese	fALS	Yes	Male	41	43	Spinal	No	UMN/LMN	No	24
5	USA	Chinese	fALS	Yes	Female	52	56	Spinal	No	LMN	No	48
6	USA	? Caucasian	?sALS	/	/	/	/	/	/	/	/	/
7	Japan	Japanese	fALS	Yes	Male	52	53	Spinal (UL+LL)	No	UMN/LMN	No	15
8	Japan	Japanese	fALS#	Yes	Male	45	47	Spinal (UL)	No	/	No	48
9	Japan	Japanese	fALS#	Yes	Male	54	/	Spinal (LL)	No	/	No	> 8
10	Japan	Japanese	fALS#	Yes	Male	/	/	/	/	/	/	/
11	Japan	Japanese	fALS	Yes	Male	54	55	Spinal (LL)	/	/	/	10
12	Hong Kong, China	Chinese	sALS	No	Female	47	49	Bulbar	/	/	/	18
13	Hong Kong, China	Chinese	sALS	No	Male	56	57	Spinal	/	/	/	10
14	Hong Kong, China	Chinese	?sALS	/	/	61-65	/	/	/	/	/	/
15	Hong Kong, China	Chinese	?sALS	/	/	41-45	/	/	/	/	/	/
16	China	Chinese	fALS	Yes	Female	43	/	Spinal (UL)	/	/	/	/
17	China	Chinese	sALS	No	/	/	/	/	/	/	/	/
18	China	Chinese	sALS	No	/	/	/	/	/	/	/	/
19	China	Chinese	sALS	No	Female	39	40	Bulbar	Yes	UMN/LMN	No	7
20	China	Chinese	sALS	No	Male	52	53	Bulbar	Yes	LMN	No	8
21	China	Chinese	sALS	No	Male	53	54	Spinal (UL)	/	UMN/LMN	No	14
22	China	Chinese	sALS	No	Male	49	51	Spinal (UL)	/	UMN/LMN	No	24
23	China	Chinese	sALS	No	Female	43	/	Spinal (UL)	/	UMN/LMN	No	/
24	China	Chinese	sALS	No	Female	38	/	Spinal (UL)	/	UMN/LMN	No	/
25	China	Chinese	sALS	No	Male	59	/	Spinal (UL)	/	UMN	No	/
26	China	Chinese	sALS	No	Male	59	/	Spinal (UL+LL)	/	UMN/LMN	No	/
27	China	Chinese	fALS	Yes	Female	73	74	Spinal (UL)	/	UMN/LMN	No	10
28	China	Chinese	fALS	Yes	Female	49	50	Bulbar	Yes	UMN/LMN	No	11
29	China	Chinese	fALS	Yes	Female	62	63	Spinal (UL)	/	UMN/LMN	No	13
30	China	Chinese	fALS	Yes	Male	46	47	Bulbar	Yes	UMN	No	14
31	China	Chinese	fALS	Yes	Female	50	51	Spinal (LL)	/	UMN/LMN	No	15
32	China	Chinese	fALS	Yes	Male	54	56	Bulbar	/	UMN/LMN	No	20
33	China	Chinese	fALS	Yes	Female	53	55	Spinal (UL)	/	UMN	No	20
34	China	Chinese	fALS	Yes	Male	50	54	Spinal (UL)	/	UMN	No	48
35	Hong Kong, China	Chinese	fALS	Yes	Female	50	52	Bulbar	Yes	LMN	No	15
36	Hong Kong, China	Chinese	fALS#	Yes	Male	around 40	40-50	/	/	/	No	/
37	Hong Kong, China	Chinese	fALS#	Yes	Male	around 40	42	/	/	/	No	/
38	Hong Kong, China	Chinese	fALS	Yes	Male	61	63	Spinal (UL+LL)	Yes	LMN	No	17
39	Hong Kong, China	Chinese	fALS	Yes	Male	59	/	?EOM	No	LMN	No	> 10
40	Hong Kong, China	Chinese	fALS#	Yes	Female	60	/	Bulbar	/	/	No	/

An ALS case with an unusual initial symptom in the EOM that facilitated pre-symptomatic genetic diagnosis was identified in our laboratory. Most ALS patients show normal cranial nerve nuclei and eye movements, with any abnormalities typically due to supranuclear deficits. EOM often remains unaffected even in long-term survivors, possibly because Wnt proteins, which are important for neuromuscular junctions, are more highly expressed. Riluzole, the only drug proven to improve survival in ALS, also enhances Wnt signaling [15]. Although diabetic neuropathy was considered a potential cause for this patient's symptoms, the unilateral pain typically present in diabetic ophthalmoplegia due to microvascular ischemia was not observed in this relatively young patient with impaired fasting glucose only and no other microvascular complications [16]. Moreover, a post-viral cause was unlikely given the absence of viral illness prior to symptom onset. However, idiopathic abducens nerve palsy, a diagnosis by exclusion, cannot be completely ruled out. Nevertheless, given the strong family history and relevant electromyography findings, ALS was diagnosed early, with treatment starting before noticeable muscle weakness. Early pre-symptomatic administration of riluzole may help slow disease progression [17], though it is unlikely to result in a complete symptom resolution. In this scenario, the patient's ocular findings might indicate a different clinical issue. However, they remained significant in leading to the subsequent identification of the *TARDBP* p.G298S mutation, typically associated with rapid disease progression, and in facilitating early multidisciplinary intervention, including pulmonary, nutritional, and psychiatric evaluations.

The phenotypes of patients with the *TARDBP* p.G298S mutation are similar to those in a review of 267 mostly Italian and Chinese patients with *TARDBP* mutations. Both groups commonly have spinal onset starting in the upper limbs and rarely show extrapyramidal signs or cognitive impairment [6]. However, the p.G298S mutation is linked to an earlier average age of onset (51 vs. 54 years) and shorter disease duration (18 months vs. 38 months). Research with TDP-43 knock-in mice indicates that *TARDBP* mutations can have incomplete penetrance, suggesting that other environmental or genetic factors may affect TDP-43 pathology and the onset of neurodegeneration [5], which might explain why no neurological manifestations were reported in the parents of probands in both families. Our findings confirm the rapid progression previously reported for the *TARDBP* p.G298S mutation and highlight its diverse phenotypic spectrum, potentially including rare presentations with EOM involvement.

Epidemiology and genetic mutations reported locally for ALS

Articles on the local epidemiology and genetic spectrum of ALS are limited; the earliest study, from 1996, reported an incidence of 0.31 per 100,000 per year and a prevalence of 0.95 per 100,000 [18]. A decade later, another study showed a significant increase in both rates to 0.60 and 3.04 per 100,000, respectively, attributing this rise to longer life expectancy, which allowed more people to reach ages when ALS typically occurs [2]. Given that two decades have passed since these studies, updated research is needed to provide a clearer picture of ALS in the region, as the current estimates from the local registry rely on outdated data. A summary of ALS-associated mutations in Hong Kong shows 42 mutations across 59 alleles (Table 4). The most common variants identified are *SOD1* p.I150T and *TARDBP* p.G298S, with five and two patients carrying these mutations, respectively, from single families [1,12]. When only probands are counted, *TARDBP* p.G298S accounts for 10.2% (7 out of 59) of all alleles, which might be due to its founder effect in this locality.

**Table 4:** Summary of the 42 ALS-associated mutations reported in Hong Kong ALS: amyotrophic lateral sclerosis [1,12]

Gene	Variant	Number of alleles
ALS2	p.L520F	1
APEX1	p.G8R	1
APEX1	p.E110G	1
ARHGEF28	p.A168T	1
ARHGEF28	p.A717V	1
ARHGEF28	p.G1029W	1
ARHGEF28	p.M1261T	1
ATXN2	p.A1023V	1
C9orf72	G4C2 repeat expansion	1
C9orf72	p.G465R	1
DAO	p.P103L	2
DAO	p.T269I	1
DCTN1	p.D1199N	2
DCTN1	p.G1013A	1
DCTN1	p.Q701R	1
DCTN1	p.A457T	1
ITPR2	p.M2443T	1
ITPR2	p.V1952A	1
KIFAP3	p.F694L	1
NEFH	p.T642M	1
NEK1	p.P287A	1
NEK1	p.L413P	1
NEK1	p.P287A	2
PLCD1	p.M412R	1
PON2	p.S31F	1
PRPH	p.L118M	2
SETX	p.M2324I	1
SETX	p.N1100S	1
SETX	p.M627T	1
SOD1	p.D93G	1
SOD1	p.I150T	8
SPG11	p.L1982S	1
SPG11	p.P194L	1
SQSTM1	p.V144I	1
SQSTM1	p.G411S	1
TARDBP	p.G298S	7
TARDBP	p.M337V	1
TARDBP	p.S375G	1
TBK1	p.L94S	1
TBK1	p.H336R	1
UNC13A	p.P960S	1
VCP	p.G157R	1
		59

Gene-silencing therapy with tofersen, an antisense oligonucleotide (ASO) targeting *SOD1*, was approved by the U.S. Food and Drug Administration for *SOD1*-associated ALS in April 2023. A trial is also ongoing to assess the safety and efficacy of Jacifusen, an ASO targeting the *FUS* mutation. Studies on therapies for *C9orf72* repeat expansion-associated ALS have not yet shown clear benefits for patients with ALS or FTD. However, these efforts have demonstrated that clinical trials for new treatments are possible, and patients are eager to participate in research. [19]. As a result, genetic testing is now recommended for all newly diagnosed ALS patients, regardless of family history or age, to facilitate access to therapeutic trials and emerging gene-based therapies [3]. In August 2023, updated guidelines for genetic testing and counseling were released to standardize practices among neurologists and other healthcare providers caring for patients with ALS. It is recommended that all ALS patients undergo genetic testing, including a *C9orf72* assay and sequencing for *SOD1*, *FUS*, and *TARDBP*, at a minimum [20].

## Conclusions

Timely ALS diagnosis is crucial for initiating disease-modifying treatments and multidisciplinary care, as advocated by local patient support groups, which can enhance quality of life and survival, especially for *TARDBP* p.G298S, which is a common mutation in the locality with a rapid disease progression. Clinicians need to recognize ALS early and expedite the diagnostic process to potentially slow down neurodegeneration potentially. This article highlighted the distinct clinical manifestations and genetic backgrounds of local ALS patients. It could be useful for developing genetic screening and counseling strategies in Hong Kong and southern China.

## References

[1] Pang SY, Hsu JS, Teo KC (2017). Burden of rare variants in ALS genes influences survival in familial and sporadic ALS. Neurobiol Aging.

[2] Fong GC, Cheng TS, Lam K (2005). An epidemiological study of motor neuron disease in Hong Kong. Amyotroph Lateral Scler Other Motor Neuron Disord.

[3] Dharmadasa T, Scaber J, Edmond E, Marsden R, Thompson A, Talbot K, Turner MR (2022). Genetic testing in motor neurone disease. Pract Neurol.

[4] Zou ZY, Zhou ZR, Che CH, Liu CY, He RL, Huang HP (2017). Genetic epidemiology of amyotrophic lateral sclerosis: a systematic review and meta-analysis. J Neurol Neurosurg Psychiatry.

[5] Xu F, Huang S, Li XY (2022). Identification of TARDBP Gly298Ser as a founder mutation for amyotrophic lateral sclerosis in Southern China. BMC Med Genomics.

[6] Lombardi M, Corrado L, Piola B (2023). Variability in clinical phenotype in TARDBP mutations: amyotrophic lateral sclerosis case description and literature review. Genes (Basel).

[7] Li J, Liu Q, Sun X (2022). Genotype-phenotype association of TARDBP mutations in Chinese patients with amyotrophic lateral sclerosis: a single-center study and systematic review of published literature. J Neurol.

[8] Nozaki I, Arai M, Takahashi K (2010). Familial ALS with G298S mutation in TARDBP: a comparison of CSF tau protein levels with those in sporadic ALS. Intern Med.

[9] Van Deerlin VM, Leverenz JB, Bekris LM (2008). TARDBP mutations in amyotrophic lateral sclerosis with TDP-43 neuropathology: a genetic and histopathological analysis. Lancet Neurol.

[10] Murakami A, Koga S, Sekiya H (2022). Old age amyotrophic lateral sclerosis and limbic TDP-43 pathology. Brain Pathol.

[11] Nakamura R, Sone J, Atsuta N (2016). Next-generation sequencing of 28 ALS-related genes in a Japanese ALS cohort. Neurobiol Aging.

[12] Yu AC, Yim AK, Chan AY (2019). A targeted gene panel that covers coding, non-coding and short tandem repeat regions improves the diagnosis of patients with neurodegenerative diseases. Front Neurosci.

[13] Lin J, Chen W, Huang P, Xie Y, Zheng M, Yao X (2021). The distinct manifestation of young-onset amyotrophic lateral sclerosis in China. Amyotroph Lateral Scler Frontotemporal Degener.

[14] Chen W, Xie Y, Zheng M, Lin J, Huang P, Pei Z, Yao X (2020). Clinical and genetic features of patients with amyotrophic lateral sclerosis in southern China. Eur J Neurol.

[15] McLoon LK, Harandi VM, Brännström T, Andersen PM, Liu JX (2014). Wnt and extraocular muscle sparing in amyotrophic lateral sclerosis. Invest Ophthalmol Vis Sci.

[16] Al Kahtani ES, Khandekar R, Al-Rubeaan K, Youssef AM, Ibrahim HM, Al-Sharqawi AH (2016). Assessment of the prevalence and risk factors of ophthalmoplegia among diabetic patients in a large national diabetes registry cohort. BMC Ophthalmol.

[17] Aggarwal A (2017). Potential therapeutic benefits of riluzole in pre-symptomatic familial amyotrophic lateral sclerosis. Ann Neurodegener Dis.

[18] Fong KY, Yu YL, Chan YW (1996). Motor neuron disease in Hong Kong Chinese: epidemiology and clinical picture. Neuroepidemiology.

[19] Wang H, Guan L, Deng M (2023). Recent progress of the genetics of amyotrophic lateral sclerosis and challenges of gene therapy. Front Neurosci.

[20] Roggenbuck J, Eubank BH, Wright J, Harms MB, Kolb SJ (2023). Evidence-based consensus guidelines for ALS genetic testing and counseling. Ann Clin Transl Neurol.

